# The chicken IL-1 family: evolution in the context of the studied vertebrate lineage

**DOI:** 10.1007/s00251-014-0780-7

**Published:** 2014-05-27

**Authors:** Mark S. Gibson, Pete Kaiser, Mark Fife

**Affiliations:** 1 The Pirbright Institute, Ash Road, Pirbright, Surrey, GU24 0NF UK; 2 The Roslin Institute and R(D)SVS, University of Edinburgh, Easter Bush, Midlothian, EH25 9RG UK

**Keywords:** Interleukin-1, Interleukin-1 receptor, Interleukin-36, Evolution, Synteny, Chicken

## Abstract

The interleukin-1 gene family encodes a group of related proteins that exhibit a remarkable pleiotropy in the context of health and disease. The set of indispensable functions they control suggests that these genes should be found in all eukaryotic species. The ligands and receptors of this family have been primarily characterised in man and mouse. The genomes of most non-mammalian animal species sequenced so far possess all of the IL-1 receptor genes found in mammals. Yet, strikingly, very few of the ligands are identifiable in non-mammalian genomes. Our recent identification of two further IL-1 ligands in the chicken warranted a critical reappraisal of the evolution of this vitally important cytokine family. This review presents substantial data gathered across multiple, divergent metazoan genomes to unambiguously trace the origin of these genes. With the hypothesis that all of these genes, both ligands and receptors, were formed in a single ancient ancestor, extensive database mining revealed sufficient evidence to confirm this. It therefore suggests that the emergence of mammals is unrelated to the expansion of the IL-1 family. A thorough review of this cytokine family in the chicken, the most extensively studied amongst non-mammalian species, is also presented.

**Electronic supplementary material:**

The online version of this article (doi:10.1007/s00251-014-0780-7) contains supplementary material, which is available to authorized users.

## Introduction

In humans, the IL-1 family contains 11 ligand genes (Table 1) encoded at three separate loci. Nine of these are present at a single locus on chromosome 2, whereas IL-18 and IL-33 lie on chromosomes 11 and 9, respectively. The ligands were originally named IL-1 F1-F11; however, this nomenclature has recently been revised ((Dinarello et al. 2010); see Table 1). Ligand members have a broad and sometimes overlapping range of prominent roles in innate and adaptive immune responses. They are thought to have arisen following gene duplication, which is reflected in their many shared characteristics. The specific bioactivities, expression, regulation and genomic organisation of these 11 ligands have been comprehensively characterised in mammals (Boraschi et al. 2011; Dinarello 2009; Dinarello 2011; Towne and Sims 2012; van de Veerdonk et al. 2012; Vigne et al. 2012).

**Table 1:** The IL-1 ligand gene family in humans

Gene name	Alternative names	Biological function	Genomic location
Interleukin-1α	IL-1A, IL1, IL-1α, IL1F1	Agonist	2: 113,531,492–113,542,167
Interleukin-1β	IL-1B, IL-1β, IL1F2	Agonist	2: 113,587,328–113,594,480
Interleukin-1 receptor antagonist	ICIL-1RA, IL-1RN, IL1F3, IL1RA, IRAP, MGC10430	Receptor antagonist	2: 113,864,791–113,891,593
Interleukin-18	IGIF, IL-18, IL-1 g, IL1F4	Agonist	11: 112,013,974–112,034,840
Interleukin-36 receptor antagonist	FIL1, FIL1δ, FIL1D, IL-1 F5, IL1F5, IL1HY1, IL1L1, IL1RP3, IL-36RN, IL36RA, MGC29840	Receptor antagonist; anti-inflammatory	2: 113,816,215–113,822,325
Interleukin-36α	FIL1, FIL1E, IL-1 F6, IL1ε, IL1F6, IL-36A, IL-36α, MGC129552, MGC129553	Agonist	2: 113,763,038–113,765,621
Interleukin-37	FIL1, FIL1ζ, FIL1Z, IL-1 F7, IL-1H4, IL-1RP1, IL1F7, IL-37	Anti-inflammatory	2: 113,670,548–113,676,459
Interleukin-36β	FIL1, FILIη, IL-1 F8, IL-1H2, IL1-ETA, IL1F8, IL1H2, MGC126880, MGC126882, IL-36B, IL-36β	Agonist	2: 113,779,668–113,810,444
Interleukin-36γ	IL-1 F9, IL-1H1, IL-1RP2, IL1E, IL1F9, IL1H1, IL-36G, IL-36γ	Agonist	2: 113,730,780–113,743,242
Interleukin-38	FKSG75, IL-1 F10, IL-1HY2, IL1-theta, MGC11983, MGC119832, MGC119833, IL-38	Receptor antagonist	2: 113,825,547–113,833,427
Interleukin-33	C9orf26, DKFZp586H0523, DVS27, IL1F11, NF-HEV, IL-33	Agonist	9: 6,215,805–6,257,983

The biological effects of the IL-1 ligands are mediated by members of the IL-1 receptor (IL-1R) family which are expressed on the surface of target cells or secreted as soluble receptors. In mammals, the family has 11 members (Table 2) that are characterised by an IgG-like extracellular domain and a cytoplasmic Toll/IL-1R (TIR) domain. The IL-1R family is part of a wider superfamily of TIR domain-containing receptors which includes the Toll-like receptors, intracellular adaptor molecules (such as MyD88), which are involved in signalling and pattern recognition, and the Toll proteins of *Drosophila*.

**Table 2:** The IL-1 receptor gene family in humans and chickens. Six members of the family are found at a locus that has conserved synteny between the two species

Gene	Genomic location	
	Human	Chicken
IL-1RII	2: 102,608,306–102,645,006	1: 133,109,396–133,119,021
IL-1RI^a^	2: 102,681,004–102,796,334	1: 133,163,682–133,185,156
IL-1RL2	2: 102,803,433–102,856,462	1: 138,039,834–138,053,899
ST2	2: 102,927,962–102,968,497	1: 133,190,561–133,209,036
IL-18Rα	2: 102,927,989–103,015,218	1: 133,259,701–133,278,851
IL-18Rβ	2: 103,035,149–103,069,025	1: 133,283,645–133,298,556
SIGIRR	11: 405,716–417,455	5: 1,555,415–1,560,214
TIGIRR1	X: 103,810,996–105,012,102	4: 16,972,376–17,142,642
TIGIRR2	X: 28,605,516–29,974,840	1: 115,540,657–115,874,541
IL-1RAcP	3: 190,231,840–190,375,843	9: 13,337,288–13,367,409
TILRR	9: 14,734,664–14,910,993	Z: 31,576,518–31,643,574

^a^The recently identified IL-1R3 (Qian et al. 2012) is a truncated IL-1RI transcript formed through the use of an internal promoter

In this review, we discuss our findings from an analysis of the chicken IL-1 family. Moreover, we consider, alongside a genomic analysis of other species, the significance of these data with regard to the evolution of IL-1 in vertebrates.

## The chicken IL-1 family

Compared with mammals, far fewer members of the IL-1 family have been identified in the chicken. To date, the ligands IL-1β, IL-1RN, IL-36RN and IL-18, as well as the receptors IL-1RI and ST2, are the only chicken orthologues that have been cloned. Analysis of chicken SIGIRR messenger RNA (mRNA) expression has been carried out by northern blot but the complementary DNA (cDNA) has not been cloned. Chicken orthologues of IL-18Rα, IL-1RAcP and TIGIRR-1 have also been identified from expressed sequence tag (EST) libraries, but have not been fully characterised.

### Chicken IL-1β

The first chicken IL-1 (chIL-1) ligand to be identified was chIL-1β, and its cDNA was cloned from lipopolysaccharide (LPS)-stimulated HD11 (an avian macrophage cell line) cells (Weining et al. 1998). It encodes a predicted protein of 267 amino acids which has 25 % identity with its human orthologue and contains an NH_2_-terminal pro-domain. Mammalian IL-1β is synthesised as a biologically inactive molecule containing a pro-domain (Jobling et al. 1988), which is cleaved by caspase-1 at a conserved aspartic acid residue (Black et al. 1988) facilitating secretion. Caspase-1 activation is dependent upon assembly of the NALP3 inflammasome (Agostini et al. 2004). The chicken orthologue of caspase-1 has also been cloned (Johnson et al. 1998), suggesting that a mechanism for chIL-1β (and chIL-18) maturation similar to that in mammals may be used. Unlike mammalian IL-1β, the chicken protein lacks a conserved aspartic acid at the predicted caspase-1 cut site. Purified recombinant mature chIL-1β (lacking the predicted pro-domain) exhibited biological activity which resembled that of its mammalian orthologues. In CEC-32 cells (a quail fibroblast cell line) stimulated with rchIL-1β, a dose-dependent increase in CXCLi1 (K60) expression was detected. Similar to mammalian IL-1β, an instability element (ATTTA) was identified in the 3′ untranslated region (UTR) of the chIL-1β transcript (Weining et al. 1998). Compared with full-length chIL-1β and three alternatively truncated forms, an N-terminal truncation mutant of chIL-1β starting immediately 5′ to Ala^106^ (the first residue after the predicted caspase-1 cleavage site) exhibited significantly enhanced (100-fold) bioactivity (Gyorfy et al. 2003). This suggests that processing is required for maximal chIL-1β activity, possibly mediated by caspase-1. A recent study has shown that chIL-1β can be processed at three alternative aspartic acid residues (D^77^, D^80^, D^82^) with sea bass caspase-1 (Reis et al. 2012). Using site-directed mutagenesis, the genuine caspase-1 cut site was confirmed as D^80^. Both human and sea bass caspase-1 cleave chicken pro-IL-1β at this position (Reis et al. 2012). In mammals, caspase-1-independent processing of IL-1β is carried out by several neutrophil proteases (Dinarello 2011). Adjacent to the caspase-1 site, single tyrosine and alanine residues have been recognised as the protease cut sites in mammals (Dinarello 2011). Although avian equivalents of the neutrophil proteases have yet to be identified in heterophils (counterpart of mammalian neutrophils), their putative cut sites are both conserved in chIL-1β.

Expression of chIL-1β is increased in response to bacterial, viral and parasite challenge, consistent with its role as a rapidly induced pro-inflammatory mediator. In IFN-γ-primed heterophils, stimulation with *Salmonella* Enteritidis led to statistically significant increases in IL-1β expression (Kogut et al. 2005). Similarly, in chicken embryonic fibroblasts (CEF), kidney cells and HD11 cells, stimulation with *Salmonella*-derived flagellin induced significant increases in IL-1β expression in all three cell types (Iqbal et al. 2005). IL-1β expression is also increased in bursal cells from IBDV-infected chickens (Eldaghayes et al. 2006) as well as in HD11 cells stimulated with TLR7 agonists (Philbin et al. 2005). Intraepithelial lymphocytes removed from the jejunum of *Eimeria maxima*-infected chickens contain high levels of IL-1β mRNA compared with uninfected controls (Hong et al. 2006).

The structure of the chIL-1β gene has been elucidated (Kaiser et al. 2004). The exon-intron structure of mammalian IL-1β is conserved in the chicken; however, the overall size of the chicken gene is substantially smaller than the human orthologue due to much shorter introns throughout. The crystal structure of chIL-1β has recently been resolved (Cheng et al. 2011), revealing that the β-trefoil conformation of the human cytokine is conserved in the chicken. Significant differences between the chicken and human structures were found in the regions involved in receptor binding, providing a molecular explanation for the inability of these cytokines to cross-react.

### Chicken IL-18

A full-length chicken IL-18 open reading frame (ORF) was identified in a bursal EST library and subsequently cloned from LPS-stimulated HD11 cells (Schneider et al. 2000). The full-length predicted protein contains 199 amino acids (aa), has 30 % identity with mammalian chIL-18 sequences and includes a pro-domain at the NH_2_-terminal. When aligned with mammalian sequences, a conserved aspartic acid is apparent at the predicted caspase-1 cleavage site, suggesting that it may be processed by the enzyme. Purified recombinant mature chIL-18 (lacking the pro-domain) exhibited biological activity similar to that of mammalian IL-18. In primary chicken splenocytes stimulated with rchIL-18, a dose-dependent increase in IFN-γ production was detected (Schneider et al. 2000). A conserved Th1 cell lineage similar to the one in mammals was subsequently proposed in the chicken. In chicken CD4^+^ splenocytes stimulated with chIL-18, cell proliferation, IFN-γ production and MHC class II expression were all increased (Gobel et al. 2003). This was dependent upon the presence of macrophages in the culture (Gobel et al. 2003).

Elevated levels of IL-18 mRNA have been detected in the spleen of birds infected with Marek’s disease virus (Kaiser et al. 2003), characteristic of a pro-inflammatory response to viral infection. IL-18 expression was also increased in the spleen of birds injected with *Salmonella* Typhimurium LPS (Sijben et al. 2003) and the heterophils of chickens treated with corticosterone (Shini et al. 2010), reflecting the typical pro-inflammatory role of this cytokine.

### Chicken IL-1RN

Interleukin-1 receptor antagonist (IL-1RN) is a naturally occurring receptor antagonist that inhibits IL-1-mediated inflammation. In mammals, two major structural variants of this gene, secretory and intracellular, are formed through alternative splicing. The secretory variant limits the bioactivity of IL-1α and IL-1β through cell surface (type I IL-1) receptor blocking, whilst the intracellular isoforms, although clearly antagonistic, may function through any of three inadequately defined mechanisms. Firstly, icIL-1RN may suppress intracellular signalling in a non-classical (non-IL-1R-dependent) manner (Banda et al. 2005). Secondly, icIL-1RN may compete with IL-1α in the nucleus to inhibit the effects of the agonist (Merhi-Soussi et al. 2005). Thirdly, icIL-1RN isoforms may be released from cells and bind to membrane-bound IL-1RI to limit IL-1 activity, in a similar way to sIL-1RN (Corradi et al. 1995; Evans et al. 2006; Levine et al. 1997; Yoon et al. 1999). The gene for IL-1RN can been found in the genome of over 30 mammalian species and, for years, remained undiscovered in the non-mammalian lineage. However, given the potency of IL-1β in immune responses, the identification of chIL-1β (Weining et al. 1998) suggested that IL-1RN would also be present in this species. Although absent from every assembled build of the chicken genome thus far, transcripts corresponding to both secretory and intracellular variants of chIL-1RN were identified from the NCBI EST database and subsequently cloned from LPS-stimulated HD11 cells (Gibson et al. 2012a). The sIL-1RN and icIL-1RN coding region cDNAs encode predicted proteins of 173 and 163 aa, respectively, although sIL-1RN contains a predicted 17 aa signal peptide, so its predicted secreted mature protein is 156 aa. Chicken sIL-1RN is very similar in length to human sIL-1RN, sharing 38.3 % aa identity, whilst chicken icIL-1RN, most similar to the human icIL-1RN1 isoform, has 38.2 % aa identity with the human equivalent (Gibson et al. 2012a). The human IL-1RN gene consists of six exons, which, through differential splicing of the first three exons (ic1, ic2 and s1), creates three different transcripts: sIL-1RN, icIL-1RN1 and icIL-1RN2. The structure of the chIL-1RN gene is similar to its human orthologue, but is comprised of five exons, which, when translated, result in isoforms very similar in size to the corresponding human proteins. The introns of the chIL-1RN gene, however, are significantly smaller than their human equivalents, resulting in the overall gene length being around one tenth that of huIL-1RN (Gibson et al. 2012a).

Both recombinant full-length chIL-1RN isoforms antagonised the IL-1β-mediated upregulation of IL-1β and iNOS genes, mirroring their function in mammals (Gibson et al. 2012a). Further splice variants of both major structural variants were also identified, which, despite being formed through well-known splicing mechanisms (exon-skipping and use of an alternative splice acceptor site) were structurally different to mammalian splice forms and yielded non-functional proteins (Gibson et al. 2012a).

Chicken IL-1RN (full-length) expression is increased in vivo following bacterial or viral infection, reflecting a typical response to that seen in experimental models of disease in mammalian species. Stimulation of three distinct monocyte/macrophage populations with LPS in vitro did not affect the expression of full-length chIL-1RN, an unexpected response that differs from previous observations in human monocytes (Gibson et al. 2012a).

### Chicken IL-36RN

Mammalian IL-36RN suppresses inflammation through its role as an IL-1RL2 (IL-1Rrp2) receptor antagonist (Debets et al. 2001; Towne et al. 2011; Vigne et al. 2011). This prevents the agonists—IL-36α, IL-36β and IL-36γ—from binding this receptor to initiate gene transcription via NF-kB and MAP kinases. Similar to IL-1RN, IL-36RN is unable to recruit IL-1RAcP on the cell surface (Towne et al. 2011). IL-36RN also interacts with the orphan receptor SIGIRR to downregulate inflammation through an as yet unelucidated mechanism (Costelloe et al. 2008). The gene for IL-36RN is present in the genome sequence of over 30 mammalian species and was previously proposed to have arisen following gene duplication from IL-1RN in the ancestral species of the mammalian lineage (Mulero et al. 2000). The chicken is the only non-mammalian species in which it has been identified to date (Gibson et al. 2012b). As with chIL-1RN, it is currently unidentifiable in the chicken genome and was uncovered through mining the NCBI EST database. Its predicted aa sequence most closely resembles IL-36RN following TBLASTN analysis of all the available mammalian genome sequences. A moderate degree of aa identity (31.2 %) between the predicted chIL-36RN sequence and human and mouse IL-36RN was found (Gibson et al. 2012b). Additional in silico analyses supported the BLAST results to unequivocally establish its identity. Phylogenetic analysis was able to confirm that it is distantly related to mammalian IL-36RN. Analysis of the predicted secondary structure of chIL-36RN identified 12 β-strands located in almost identical regions to those in mouse IL-36RN, thus far the only experimentally resolved IL-36RN structure (Gibson et al. 2012b). ChIL-36RN is encoded by a six-exon gene, consisting of four coding region exons with the 5′ UTR comprised of two further exons. The human orthologue shares this exonic structure; however, it has a much longer 3′ UTR and its intron sizes differ from those of the chicken gene.

Human IL-36RN appears to be processed non-classically with recent data, suggesting that proteolytic cleavage yields a mature protein with increased specific activity (Towne et al. 2011). The protein does not possess a signal peptide like IL-1RN, nor have an obvious pro-domain like IL-1β and IL-1α. Instead, the fully active protein simply lacks the NH_2_-terminal methionine; a mechanism to explain its removal remains unknown. Mammalian IL-36 cytokines exhibit maximal activity when processed exactly nine residues upstream of a conserved three aa motif. In the huIL-36RN sequence, this equates to cleavage between the start codon and an adjacent valine residue (Towne et al. 2011). Although a similar three aa motif is conserved in chIL-36RN, there is no evidence that the same mode of processing (and specific activity thereafter) is also conserved in birds, as chIL-36RN has a much longer NH_2_-terminus than the human protein.

Chicken IL-36RN mRNA expression levels were significantly increased following infection with *Salmonella* Typhimurium. However, in an infectious bursal disease virus challenge model, a striking decrease in chIL-36RN mRNA expression levels was observed. This trend was evident in birds that were either resistant or susceptible to this virus, though the largest relative differences in expression were observed between infected and uninfected susceptible birds. A role for this cytokine in response to viral challenge has yet to be documented in mammals.

Whilst all other analyses of chIL-36RN suggested that its identity had been correctly ascribed, characterisation of its bioactivity did not yield findings similar to those observed for huIL-36RN. This was principally due to the absence of IL-1RL2 agonist ligands in the chicken, which would be required to facilitate a more comprehensive assessment of function.

### Chicken IL-1RI

The type I chicken IL-1 receptor (chIL-1RI) was cloned from a primary chicken fibroblast cDNA library. When compared with its human and mouse orthologues, amino acid identity of the five major protein domains varied from 19 to 61 %, with the cytoplasmic domain the most highly conserved region (Guida et al. 1992). A bioactive form of soluble chIL-1RI (sIL-1RI) has also been cloned (Klasing and Peng 2001).

### Chicken ST2

The chicken orthologue of the ST2 receptor was cloned from cDNA isolated from chicken embryos (Iwahana et al. 2004). Three different cDNAs were identified and designated ST2, ST2L and ST2 LV. ST2 is the secreted, soluble form of the receptor of which a homologue has also been identified in humans. ST2L is a longer, membrane-bound isoform which contains a transmembrane domain, whilst ST2LV is a novel splice variant of the ST2L transcript. ST2LV lacks the transmembrane domain present in ST2L and as such is a secreted protein. To date, this novel splice variant has not been found in any mammalian species. ST2L has 39.3 and 38.3 % aa identity with its respective human and mouse orthologues. In all three species, ST2L contains a signal peptide of similar length and all six cysteine residues are conserved in the Ig-like domain region. The structures of the human and chicken ST2 genes are very similar (Iwahana et al. 2004). The ligand for this receptor, IL-33, has yet to be identified in the chicken.

### Chicken SIGIRR (TIR8)

A full-length chicken SIGIRR transcript with 86 % nucleotide identity with human SIGIRR was identified in the NCBI nucleotide database (Riva et al. 2009). Northern blotting analysis of 21 different chicken tissues revealed that the expression was ubiquitous with relatively high levels in the kidney, small and large intestine, caecum, liver, glandular stomach and cloaca (Riva et al. 2009). A splice variant was identified exclusively in the adrenal gland. No further analyses of this gene have been performed.

### Other chIL-1 receptors

**Fig. 1:**
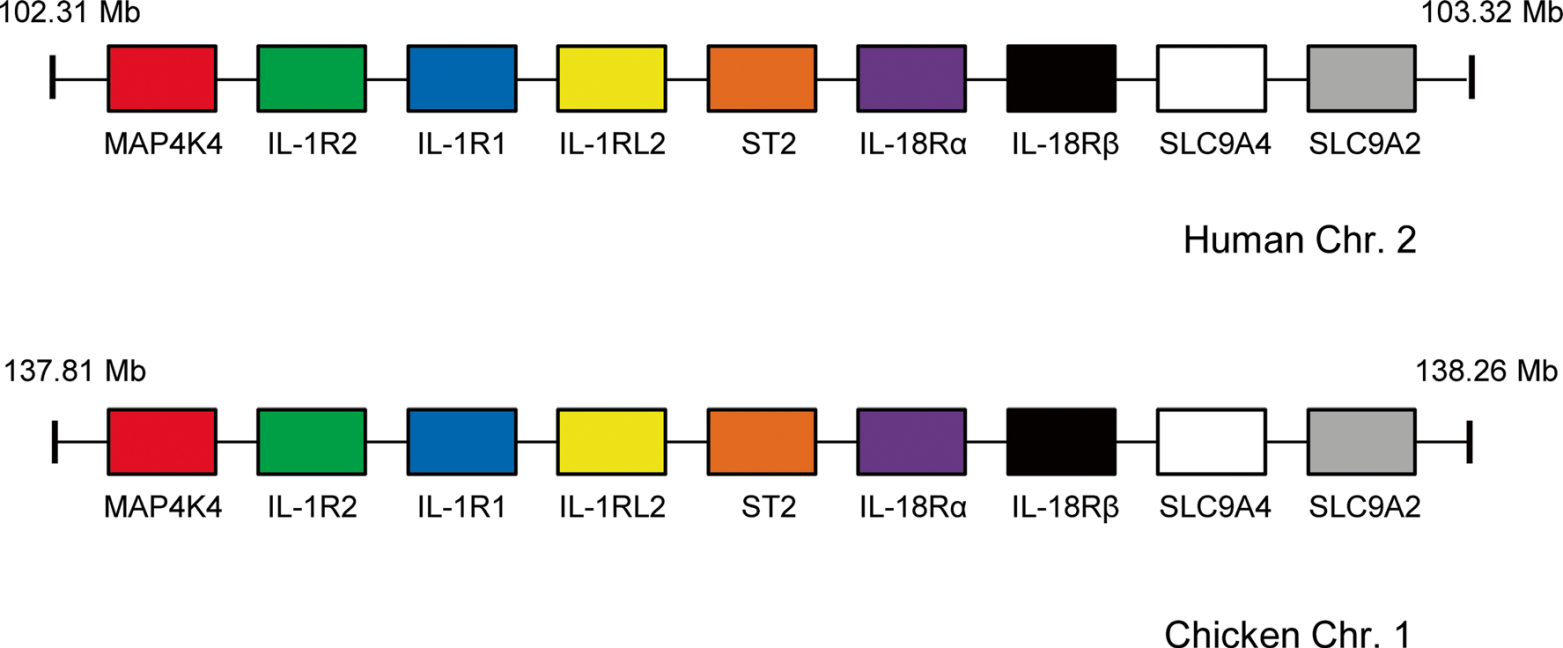
Schematic depicting the IL-1 receptor gene family locus with complete conserved synteny between humans and chickens

**Fig. 2:**
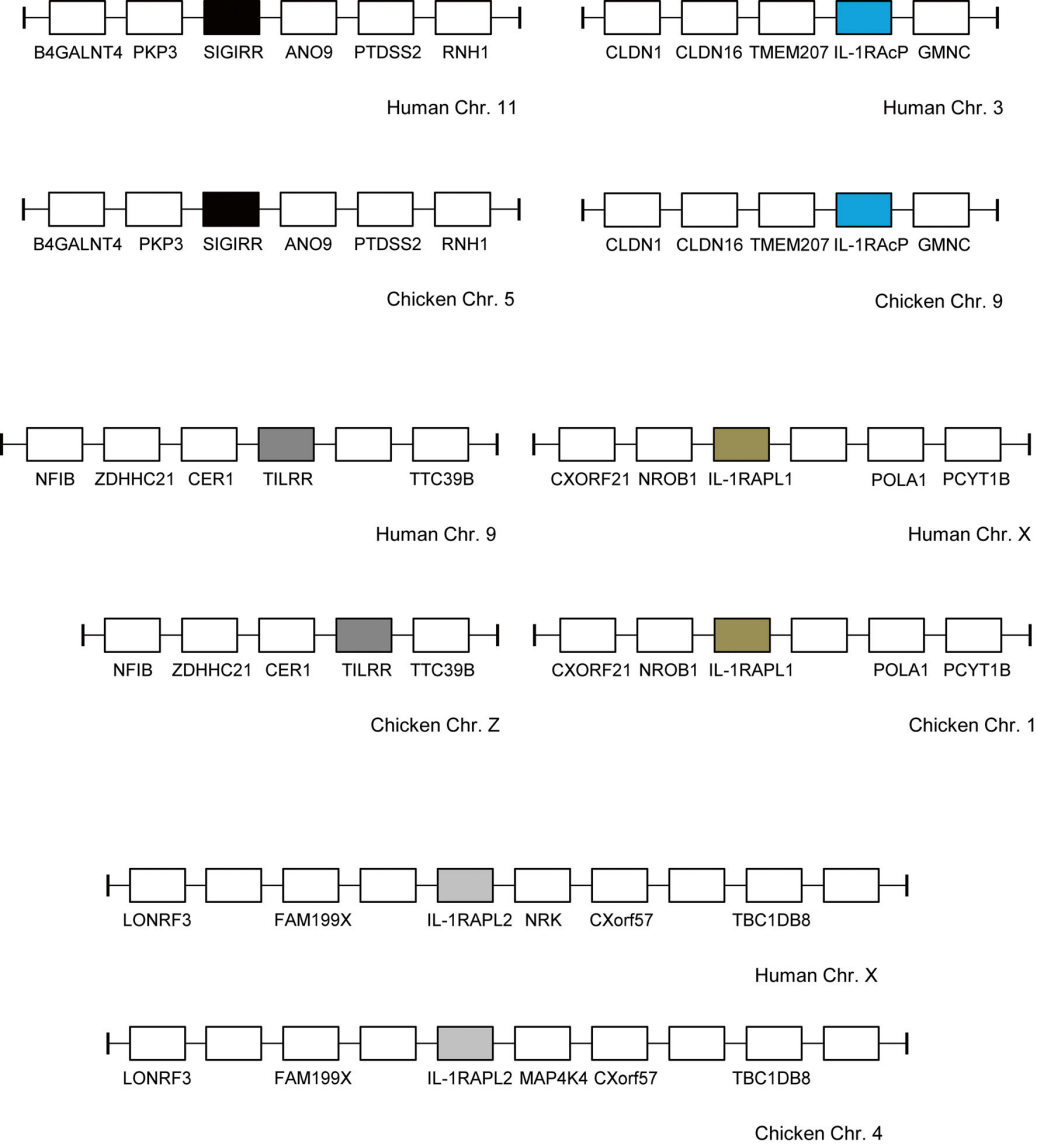
IL-1 receptor gene loci conserved between the chicken and human genomes. Direct IL-1R orthologues have identical shading. Both SIGIRR and IL-1RAcP loci lie in the reverse orientation in the chicken genome. NRK on human Chr. X is a MAP4K4 paralogue

In humans, the IL-1RII, IL-1RI, IL-1RL2, ST2, IL-18Rα and IL-18Rβ genes lie in a cluster on chromosome 2 in that order (Fig. 1). An examination of conserved synteny between this cluster and the chicken genome revealed that the huIL-1R gene cluster is fully conserved in the chicken genome on chromosome 1 (1: 133,109,396–133,298,556) (Fig. 1). Previous studies reporting the identification and cloning of chIL-1RI (Guida et al. 1992) and chST2 (Iwahana et al. 2004) failed to determine a genomic location. A partial chIL-18Rα EST had already been identified (Huising et al. 2004); however, chicken orthologues of IL-1RII and IL-18Rβ had never been described. Other members of the huIL-1R family reside elsewhere in the genome, necessitating analysis of conserved synteny between the two species to identify the remaining chIL-1R genes. The chicken gene for SIGIRR/TIR8 is on chromosome 5, at a locus that is highly conserved with the SIGIRR/TIR8 locus on human chromosome 11. Full-length chicken cDNAs of two other IL-1 receptors (TIGIRR1, TIGIRR2) and two IL-1 co-receptors (IL-1RAcP and TILRR) found in humans had never been isolated. Again, conserved synteny was used to identify these genes in the chicken. They are dispersed across the genome, as they are in humans. Human TIGIRR1/IL-1RAPL2, found on human chromosome X, has a chicken orthologue on chromosome 4 (4: 16,972,376–17,142,642). Only a partial chTIGIRR-1 EST had been previously identified (Huising et al. 2004). Human TIGIRR2/IL-1RAPL1, which is also found on chromosome X, has an orthologue in the chicken genome on chromosome 1 (1: 115,540,657–115,874,541). Human IL-1RAcP is found on chromosome 3 at a locus syntenic with a region of chicken chromosome 9, which contains the chIL-1RAcP gene (9: 13,337,288–13,367,409). A partial chIL-1RAcP EST had been previously identified (Huising et al. 2004) but unmapped in the genome. TILRR, a novel IL-1RI co-receptor recently discovered in human and mouse genomes (Zhang et al. 2010), also has a chicken orthologue. The human gene lies on chromosome 9, with the chicken gene present on chromosome Z (Z: 31,576,518–31,643,574). Syntenic loci for all five receptors are depicted in Fig. 2. Thus, our analysis has shown that all of the IL-1 receptor genes found in the human genome are also present in the chicken. By contrast, only 4 of the 11 IL-1 ligand genes present in the human genome have been identified in the chicken. This discrepancy is particularly striking when the crucial functional roles of these mammalian ligand genes (Nold et al. 2010; Schmitz et al. 2005; Towne and Sims 2012; Vigne et al. 2012) apparently “missing” in the chicken are considered.

## Evolution of the IL-1 family in vertebrates

The past decade has yielded a significant volume of nucleotide sequence data, furthering our knowledge and understanding of the repertoires of immune function genes in many vertebrates. The availability of multiple novel genome builds, improved versions of existing builds and accompanying transcriptome datasets has enabled meaningful comparisons to be made regarding the evolution of certain gene families. In two previous studies (Huising et al. 2004; Subramaniam et al. 2004), thorough phylogenetic and molecular analyses of the known members of the IL-1 family in mammals, fish and chicken were described. Here we build on these findings by focussing on a genomic analysis of the ligands and receptors across a more diverse range of species.

### Ligands

Nine of the 11 known IL-1 ligand genes are present at a single locus in the majority of sequenced mammalian genomes. Rodents, who lack IL-37 and whose IL-1α and IL-1β genes are separated from the other six genes by ~105 Mb of sequence on Chr. 2, are the only notable exception to this amongst mammals (Nicklin et al. 2002; Taylor et al. 2002). As such, when considered alongside the many other characteristics shared by these genes, it has been suggested that this gene family arose through successive gene duplications (Busfield et al. 2000; Eisenberg et al. 1991; Nicklin et al. 2002; Taylor et al. 2002).

**Fig. 3:**
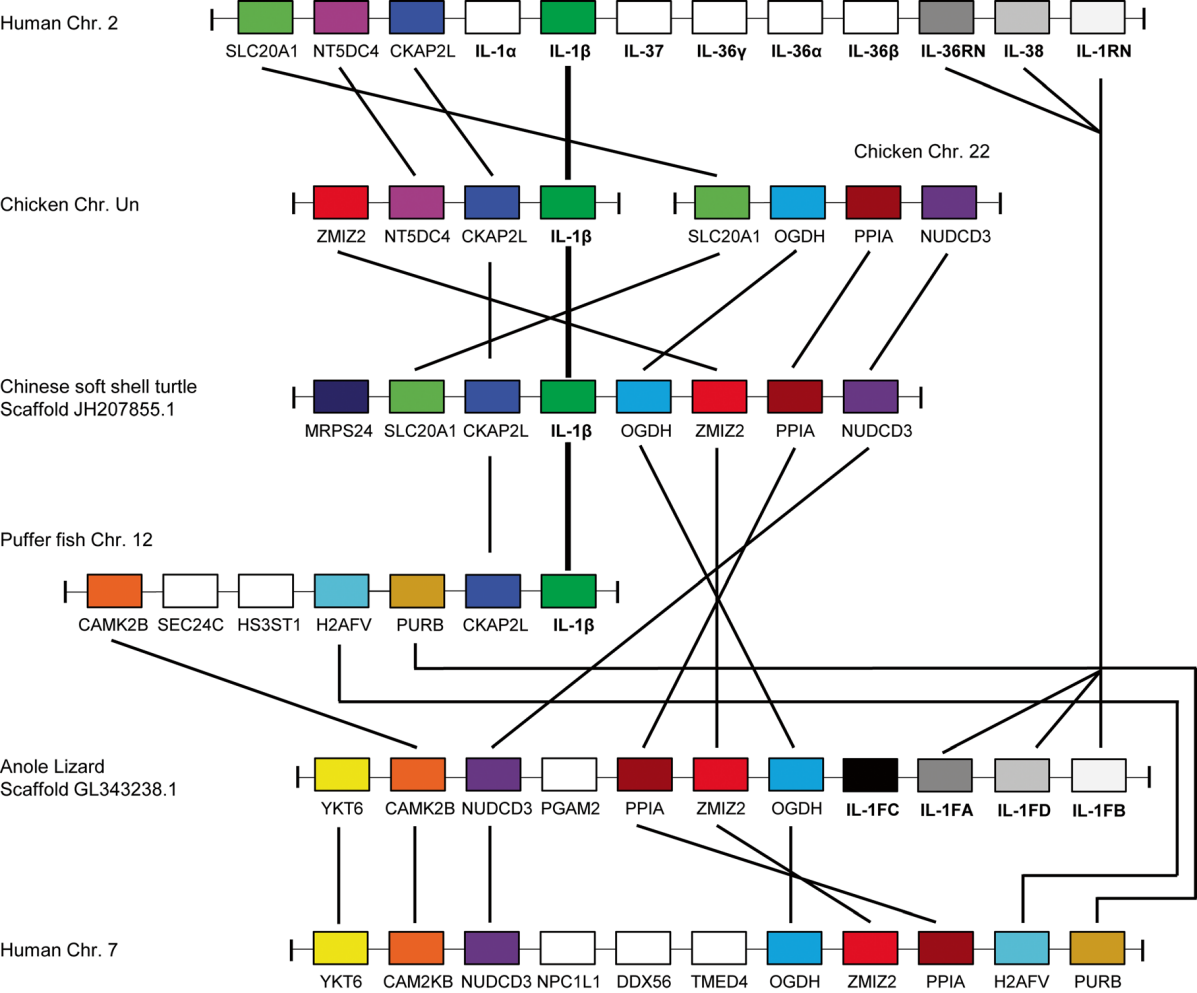
Schematic depicting an IL-1 ligand gene family locus with a degree of conserved synteny in the human, chicken, puffer fish, Chinese soft turtle and anole lizard genomes. Eight of the flanking genes found at IL-1 gene loci in non-mammalian species are conserved at a single locus on human chromosome 7. In the anole lizard, BLAST analysis (Gibson et al. 2012a) indicates IL-1FA, IL-1FB and IL-1FC are IL-1RN, IL-36RN and IL-38, though their exact identities cannot be determined with confidence. IL-1FD is homologous to IL-1FA-C; however, it is not clear which huIL-1 F orthologue it most closely resembles. Direct orthologues have identical shading

By contrast, it is not possible to make the same inference for non-mammalian species. Examination of conserved synteny between the human and chicken genome showed that the nine gene IL-1 cluster in humans is represented by only a single chicken IL-1 gene, IL-1β, at the equivalent chicken locus. Despite excellent supporting evidence from other non-mammalian species (Fig. 3), the most recent build of the chicken genome (v4.0) no longer places chIL-1β on chromosome 22. It is now unplaced in v4.0, and where it was mapped on Chr. 22 in the previous genome build (v2.1), there is now a large gap in the current build. The degree of conserved synteny with the other non-mammalian species suggests that the v2.1 build is correct at Chr. 22, and the new assembly of this chromosome is incomplete. The chicken also possesses two receptor antagonist genes, IL-1RN and IL-36RN, which have yet to be mapped in the genome but are not at the same locus as chIL-1β (Gibson et al. 2012a; Gibson et al. 2012b). This represents a major difference between the chicken and mammals. In the puffer fish, the softshell turtle and anole lizard genomes, similar conserved IL-1 gene loci exist (Fig. 3). As in the chicken, all contain fewer IL-1 genes than the mammalian IL-1 locus; however, each possesses conserved flanking genes that they share with at least three other species. The number of direct orthologues shared at these syntenic loci between this divergent group of species (Fig. 3) suggests a common origin. In fact, the position of the CKAP2L gene, which effectively “tethers” IL-1β in most mammalian and non-mammalian species (data not shown), indicates that an ancestral “founder” locus existed even before the divergence of birds and mammals over 310 million years ago.

So, has this group of non-mammalian species ever possessed (or do they still possess) the remaining “missing” genes that are seen in mammals? It is possible that the multi-IL-1 gene locus found in most mammals was formed through duplication in the mammalian lineage. Alternatively, these genes may have been present in an ancestor and have since become lost and/or dispersed in non-mammalian species. We have also previously discussed the possibility of an alternative model of gene duplication (Gibson et al. 2012a). In this model, an ancient IL-1 locus with a small number of genes (e.g. IL-1β and IL-1RN) was duplicated. Further duplications led to differential expansion of paralogous IL-1 loci in different species. Species-specific mutations and/or deletions could then have followed this to produce the IL-1 loci we now see in these different species. In our analysis of chIL-1RN, we discussed why we think it emerged following gene duplication from a common ancestor; however, we did so by only comparing the chicken and human orthologues (Gibson et al. 2012a). In this review, our analysis has now been expanded to include IL-1RN in reptiles and an IL-1β antagonist in teleost fish (Wang et al. 2009). From this, we can conclude that the presence of IL-1RN in the chicken and anole lizard as well as a teleost IL-1β antagonist gene, at different loci to IL-1β in their respective genomes, confirms that at least one ancestral IL-1 gene duplication occurred prior to a major rearrangement event. The large number of non-IL-1 direct orthologues shared between the five species in Fig. 3 suggests that this duplication preceded speciation and took place in a common ancestor. The number of confirmed structural and functional similarities between chIL-1RN and huIL-1RN (Gibson et al. 2012a) further supports this theory, i.e. that mammalian IL-1RN genes also originated from the same primordial gene and not following the emergence of mammals. Although possible, it seems implausible that all IL-1RN orthologues identified thus far have emerged as products of species-specific convergent evolution.

**Fig. 4:**
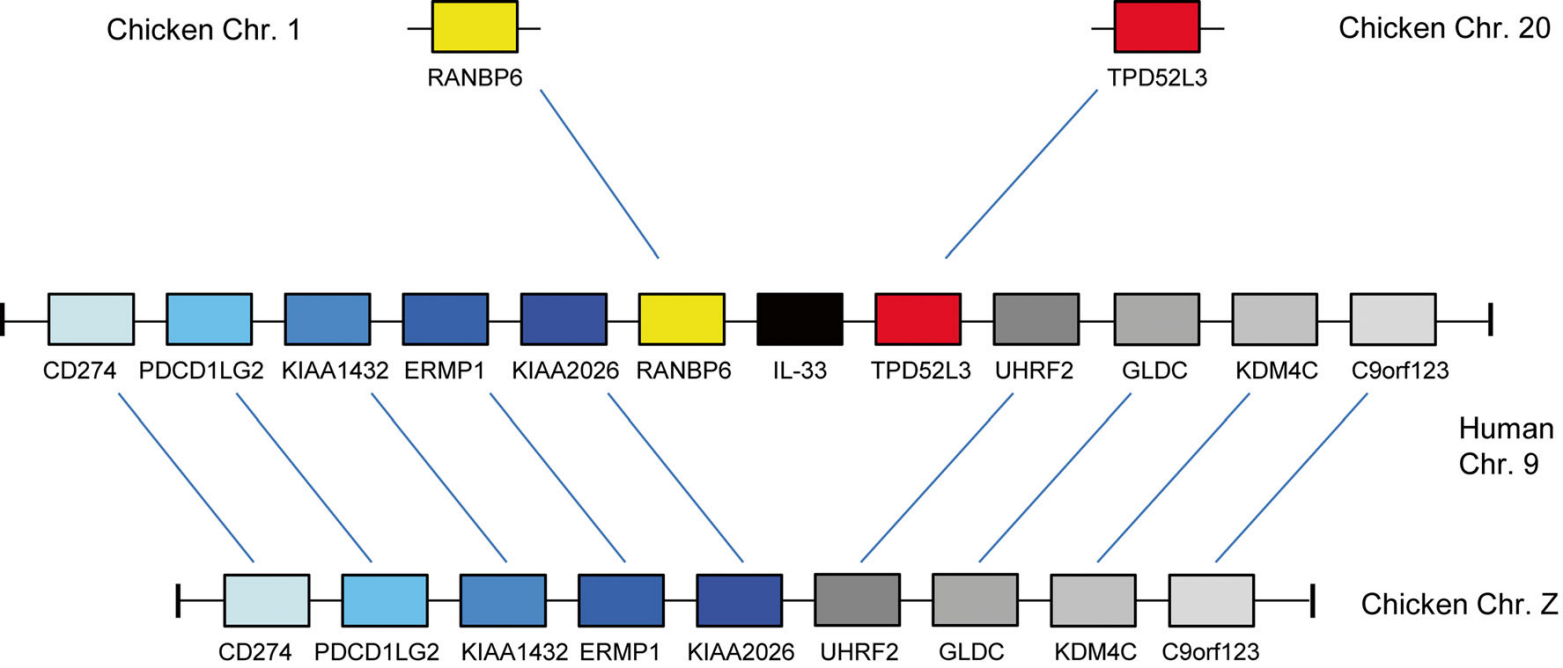
Schematic depicting the IL-33 locus in the human genome and a conserved syntenic region of the chicken genome. Avian orthologues of two of the genes (RANBP6 and TPD52L3) on human chromosome 9 lie elsewhere in the chicken genome. An IL-33 gene has yet to be identified in the chicken genome. Pairs of orthologous genes are indicated with lines and have identical shading

Recently, a small number of publications have outlined some important roles played by other IL-1 family members (Mutamba et al. 2012; Ramadas et al. 2012; van de Veerdonk et al. 2012; Vigne et al. 2011; Vigne et al. 2012). Of note, the IL-36 agonists have the capacity to shape the adaptive immune response through their effects upon CD4^+^ T cells (Vigne et al. 2011). The presence of the IL-36 receptor (IL-1RL2) and IL-36RN genes in both the chicken and anole lizard genomes strongly implies that at least one IL-36 agonist gene must have been present in the ancestral species. Both of these species currently have poorly assembled genomes with regions of low coverage and contain many large gaps in regions that are difficult to sequence. Should either or both retain IL-36 agonist genes, the analysis of their structure, function and genomic locations could strengthen the notion of a multi-IL-1 gene “founder” locus. One of the other IL-1 family ligand members yet to be identified in the chicken is IL-33, yet the gene which encodes its receptor, ST2, is conserved in the chicken genome in a location syntenic with the human orthologue. Several other non-mammals possess this receptor (data not shown). Examining conserved synteny between the human IL-33 gene locus and the chicken genome does not identify an avian orthologue. Despite this, conserved synteny between large numbers of genes flanking huIL-33 and a region on chicken chromosome Z is evident (Fig. 4). When evaluating pairings of the direct orthologues shared between both species at this locus (Fig. 4), it is clear that a chromosomal breakpoint has occurred and a potential chIL-33 gene has been lost or relocated. An examination of anole lizard, softshell turtle and tetraodon genomes shows that a number of the genes in this cluster on chicken Chr. Z are also conserved at syntenic loci in those species (data not shown). This suggests that the mammalian IL-33 locus and its conserved syntenic loci in non-mammalian species may have a shared, ancient origin. This therefore indicates that the mammalian IL-33 locus reflects the ancestral one and has probably been rearranged in non-mammalian species, should this IL-1 family member have ever existed within that lineage. Although IL-33 has only been found in mammals thus far, it was conceivably present in the common ancestor of all vertebrates when this locus was formed.

### Receptors

**Fig. 5:**
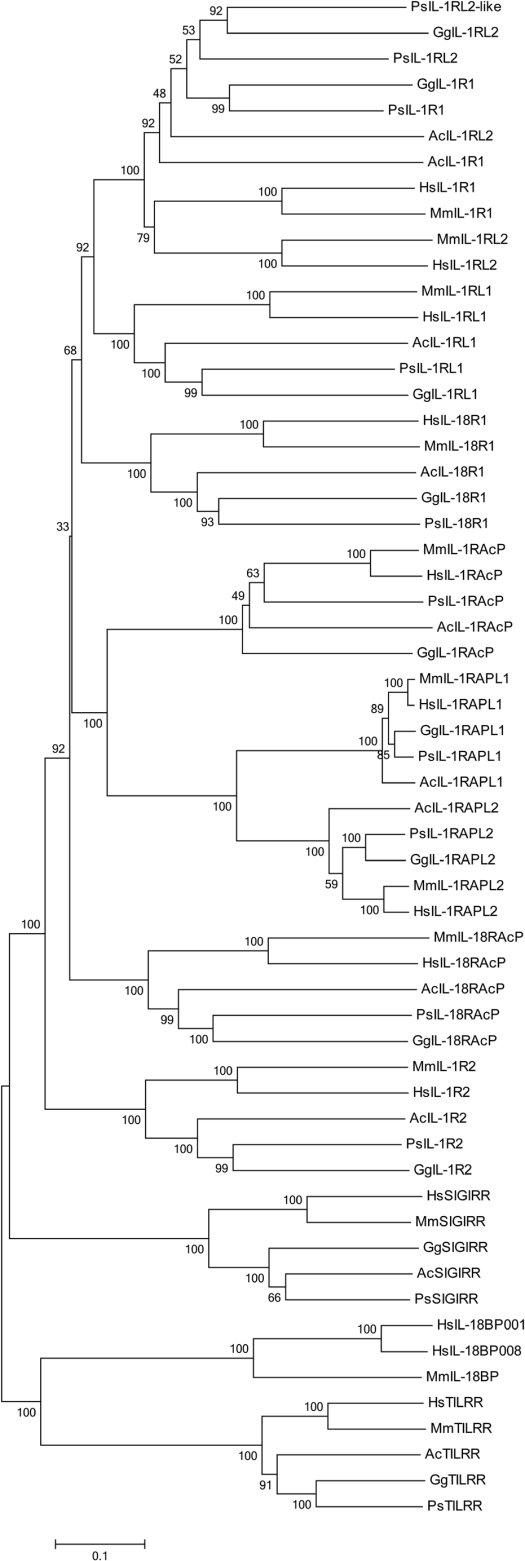
Phylogenetic analysis of the human (*Homo sapiens*: *Hs*), mouse (*Mus musculus*: *Mm*), chicken (*Gallus gallus*: *Gg*), anole lizard (*Anolis carolinesis*: *Ac*) and Chinese softshell turtle (*Pelodiscus sinensis*: *Ps*) IL-1 receptor aa sequences using MEGA v6.0. Analysis was performed using the neighbor-joining method with bootstrap analysis with 500 bootstrap datasets

The IL-1 receptor genes of mammalian and non-mammalian species are remarkably well conserved despite millions of years of divergent evolution. Several IL-1R genes with conserved TIR and extracellular Ig domains were previously analysed in mammals, chicken and fish (Huising et al. 2004; Subramaniam et al. 2004). However, a lack of genomic sequence for many of these species meant these studies, whilst comprehensive, were incomplete. With an improved build of the chicken genome now available, we observed that all of the IL-1R genes found in the human genome are present in the chicken at conserved loci. As with the IL-1 ligand genes, our analysis was extended to other non-mammalian species, which revealed a similar degree of receptor gene conservation. At the major IL-1 receptor gene locus, examining conserved synteny between the human, chicken, anole lizard and softshell turtle genomes showed that the latter two species possess all six genes found in man and birds (Supplementary Table 1). The locus is split between scaffolds in the turtle, with an additional IL-1RL2-like gene also apparent. The degree of structural similarity between IL-1R paralogues and direct orthologues (Huising et al. 2004) implies the major IL-1R gene cluster arose following successive duplication events. Furthermore, conservation at the DNA level across such a broad range of species suggests that these genes are under a strong selective pressure and likely to retain an indispensable and, between orthologues, homologous function. Phylogenetic analysis of the IL-1R genes in human, mouse, chicken, anole lizard and softshell turtle confirms the receptors in all three non-mammalian species have a common origin they share with mammals (Fig. 5). It will be interesting to determine whether intracellular signalling pathways are also conserved in each of these non-mammalian species.

**Fig. 6:**
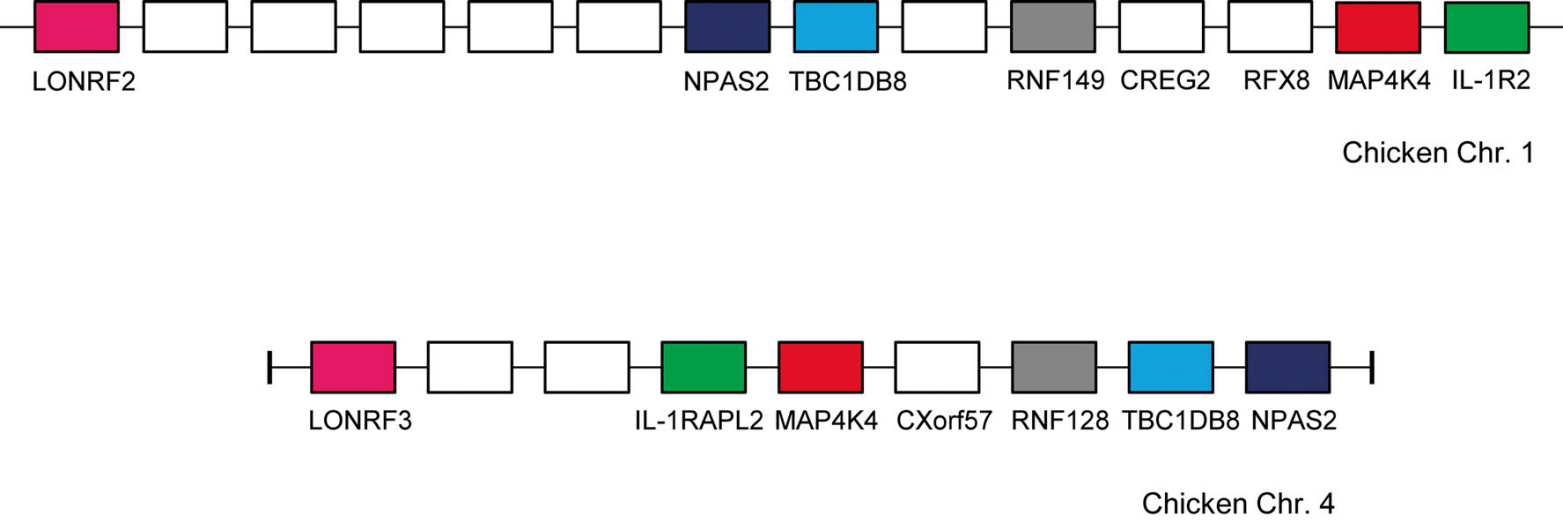
Schematic depicting paralogous loci on chromosomes 1 and 4 in the chicken genome. Direct paralogues have identical shading

Although all of the IL-1R family share sequence homology and conserved functional domains, it is interesting to note that four members are “isolated” at loci separate from the main six-gene cluster. The level of similarity across the family indicates all ten of the IL-1R genes arose from a common ancestral gene as we (and others) have proposed for the ligands. We investigated this possibility further, and report here for the first time a single observation to partially support this theory. Immediately adjacent to the IL-1R2 gene at the chicken and human major IL-1R loci is an unrelated mitogen-activated protein kinase gene (MAP4K4). This gene has a number of paralogues within the genomes of both these species, and one is found on chromosomes 4 and X in the chicken and human, respectively. This particular paralogue is directly flanked by IL-1RAPL2 (Fig. 6). To establish if this observation was restricted to a limited number of species, we scrutinised the genomes of further species and found that the same genomic structure exists in several fish, platypus, *Xenopus* and numerous mammals (data not shown). In addition to IL-1RAPL2 and MAP4K4, there are four additional unrelated genes, all of which, strikingly, possess a paralogue close to the major IL-1R gene locus (Fig. 6). Thus, there is profound evidence of duplication at a single region prior to chromosomal rearrangement and IL-1R gene duplication at the major IL-1R locus. Some of these additional unrelated genes (MAP4K4, TBC1D8, RNF149, NPAS2 and LONRF3) are also found at the IL-1RAPL2 loci in fish, platypus, *Xenopus* and other mammalian genomes (data not shown) confirming that the duplications have ancient origins.

## Concluding remarks

In this review, we utilised improved genomic resources alongside our experimental findings in the chicken to further our understanding of the evolution of the IL-1 gene family. Since this subject was last reviewed almost a decade ago (Huising et al. 2004), our work in the chicken has identified two IL-1 receptor antagonist genes for the first time in a non-mammalian species (Gibson et al. 2012a; Gibson et al. 2012b). The fact that they reside at an alternative locus to IL-1β yet retain structural and functional similarities with their mammalian orthologues suggests that the previous conjecture on the evolution of these two receptor antagonists is likely to be incorrect (Mulero et al. 2000). An IL-1β antagonist in rainbow trout (Wang et al. 2009) and three IL-1 genes in lizards that group with the chicken, human and mouse receptor antagonists (Gibson et al. 2012a) have also been identified. When considered alongside a thorough examination of the genomes of several mammalian and non-mammalian vertebrates, the most likely explanation for the evolution of IL-1 ligand genes was the formation of the family in a common ancestor that preceded speciation. These genes were then presumably subjected to significant selective pressures provided by the distinct set of challenges presented to an individual species. In the chicken, lizard and rainbow trout, this seems to have resulted in them becoming dispersed across the genome. The identification of a single locus, on human chromosome 7, containing most of the genes that flank the IL-1 ligands of non-mammals endorses our view that the family was formed before speciation.

A concurrent analysis of the receptors of this cytokine family led us to a broadly similar theory to that proposed for the ligands—that ancient gene duplications initially formed a single cohort of receptor genes. The post-speciation evolution of these receptor genes, however, differs greatly from that of the ligands. The single major ligand gene locus in mammals has remained intact but has become fragmented in the genomes of several non-mammals. The sequences of these ligands have also diverged rapidly, presumably in response to pathogen challenge, such that aa homologies are typically low (25–40 %) between orthologues. The IL-1 receptor genes of relatively distant species have remained well conserved at syntenic loci in their genomes, yet aa sequence identities are similarly low (30–42 % between humans and chickens) amongst orthologues at the major IL-1R locus. It is also interesting to observe that the aa sequence identity between the IL-1R orthologues isolated at discrete loci (SIGIRR, TIGIRR-1, TIGIRR-2 and IL-1RAcP) remains relatively high (56–95 % between humans and chickens). Conservation of the entire IL-1 receptor gene family in the three non-mammalian species studied suggests that there may be further ligand genes to be identified in each.

In conclusion, we believe we have identified evidence to show the ligands and receptors of the IL-1 cytokine family in all vertebrates may have evolved from the same ancient ancestor. Furthermore, we believe the greater number of ligand genes identified in mammals is not necessarily the product of mammalian lineage-specific gene duplication.

## Electronic supplementary material

Below is the link to the electronic supplementary material.

ESM 1 (DOCX 15 kb)
