# Supplementary material for: The chicken IL-1 family: evolution in the context of the studied vertebrate lineage
Source: Immunogenetics. 2014 May 27;66(7):427–38. doi: 10.1007/s00251-014-0780-7 (PMC4090809; doi:10.1007/s00251-014-0780-7)
Supplement: Supplementary file 1 — (DOCX 15 kb) [file 251_2014_780_MOESM1_ESM.docx]

| Species | Gene | Genomic location (scaffold)  (ENSEMBL browser 73) |
| --- | --- | --- |
| Anole lizard  (*Anolis Carolinesis*) | IL-1R2  IL-1R1  IL-1RL2  IL-1RL1 (ST2)  IL-18Rα (IL-18R1)  IL-18Rβ (IL-18RAP) | GL343243.1: 1,782,367-1,797,806  GL343243.1: 1,720,514-1,747,264  Uncurated*  GL343243.1: 1,635,929-1,658,987  GL343243.1: 1,582,854-1,609,154  GL343243.1: 1,550,668-1,573,314 |
| Softshell turtle  (*Pelodiscus sinensis*) | IL-1R2  IL-1R1  IL-1RL2  IL-1RL2-like  IL-1RL1 (ST2)  IL-18Rα (IL-18R1)  IL-18Rβ (IL-18RAP) | JH211567.1: 229,441-249,752  JH211567.1: 127,117-155,678  JH211567.1: 78,181-103,719  JH211567.1: 42,708-65,058  JH209941.1: 21,884-43,482  JH209941.1: 68,987-102,359  JH209941.1: 130,627-158,266 |

**Supplementary Table I.** IL-1 receptor genes at the major IL-1R locus in anole lizard and softshell turtle species. Genes have been identified following annotation of the scaffolds assembled from raw genomic sequence. *Though an annotated IL-1RL2 gene structure does not appear in the genome browser (ENSEMBL 73), several vertebrate IL-1RL2 cDNAs and proteins align to the reference sequence in the expected position between IL-1R1 and IL-1RL1 genes (approximate genomic coordinates: GL343243.1:1682500-1707590).
